# Supplementary material for: Mitochondrial electron transport chain, ceramide, and coenzyme Q are linked in a pathway that drives insulin resistance in skeletal muscle
Source: eLife. 2023 Dec 27;12:RP87340. doi: 10.7554/eLife.87340 (PMC10752590; doi:10.7554/eLife.87340)
Supplement: Supplementary file 2. [file elife-87340-supp2.docx]

Source

| Oligo Name | Sequence |
| --- | --- |
| rNdufa10 R | AAAGCCCGTACCTCAGCTTG |
| rNdufa12_F | CTGGGATGTGGATGGAAGCA |
| rNdufa12_R | TGTTGTTGGAGGGTCGTCAG |
| rNdufa13_F | ATGGGTTACGCACCAAGGAG |
| rNdufa13_R | ATTATGCACGTCGGCTCAGG |
| rNdufa5 F | TCTACGTTCGATTGAGCGGG |
| rNdufa5 R | CAATCCCACCAGGCCAGTAG |
| rNdufa6_F | CGGGGCTTCCTTAGCAAGAT |
| rNdufa6_F | GTCCCGACTGAAAATGGGCT |
| rNdufb3_F | GTGTCTCCTACCGCAGTCAA |
| rNdufb3_R | GCAAGGCTCCCAGACAAGAC |
| rNdufb5 F | TGTGGTTCAGGTGATGCGTT |
| rNdufb5_F | AGTTCCCAGGCCTCTAGCTT |
| rNdufv1_F | ACCTCATTTGGCTCGCTGAA |
| rNdufv1_F | CCTTCAGCCTCCAGTCATGG |
| rNdufs3_F | ATTTCCACTTCCGGTCCGTG |
| rNdufs3 R | CATGTTCCTTAGGGTGCCGA |
| rNdufb9_F | GCTTGATGAGAGCCCGGTTT |
| rNdufb9_F | TGAGGATGCTGGTTTTGCCA |
