# Supplementary figures and images for: Mitochondrial electron transport chain, ceramide, and coenzyme Q are linked in a pathway that drives insulin resistance in skeletal muscle
Source: eLife. 2023 Dec 27;12:RP87340. doi: 10.7554/eLife.87340 (PMC10752590; doi:10.7554/eLife.87340)

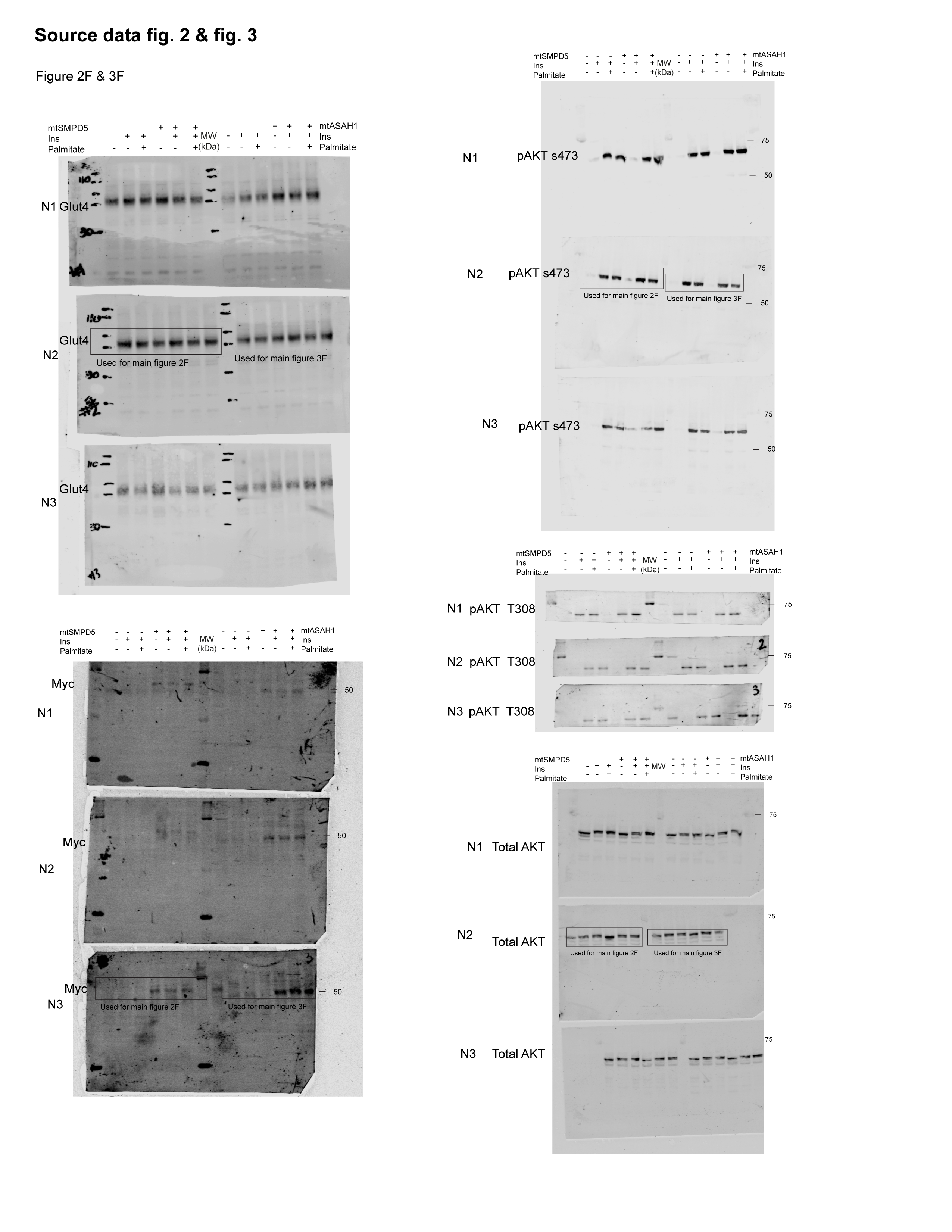

Supplement: Source data 1. [file elife-87340-data1.zip › Figure 2 and 3source data 1.tif]

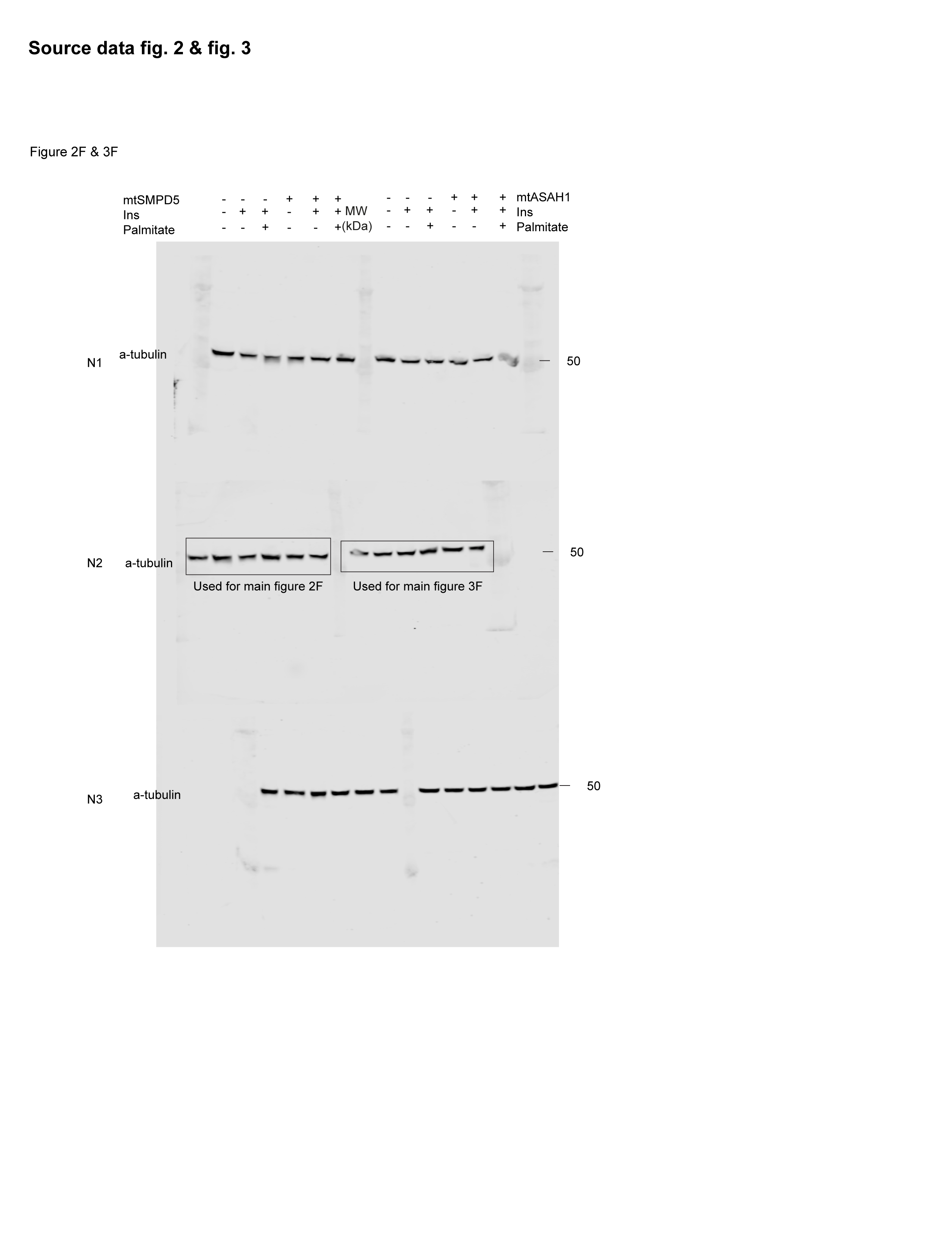

Supplement: Source data 2. [file elife-87340-data2.zip › Figure 2 and 3source data 2.tif]
